# Correlative Imaging of the Murine Hind Limb Vasculature and Muscle Tissue by MicroCT and Light Microscopy

**DOI:** 10.1038/srep41842

**Published:** 2017-02-07

**Authors:** Laura Schaad, Ruslan Hlushchuk, Sébastien Barré, Roberto Gianni-Barrera, David Haberthür, Andrea Banfi, Valentin Djonov

**Affiliations:** 1 Institute of Anatomy, University of Bern, Baltzerstrasse 2, 3012 Bern, Switzerland; 2 Graduate School for Cellular and Biomedical Sciences, University of Bern, Switzerland; 3 Department of Biomedicine, University Hospital Basel, Hebelstrasse 20, 4031 Basel, Switzerland

## Abstract

A detailed vascular visualization and adequate quantification is essential for the proper assessment of novel angiomodulating strategies. Here, we introduce an *ex vivo* micro-computed tomography (microCT)-based imaging approach for the 3D visualization of the entire vasculature down to the capillary level and rapid estimation of the vascular volume and vessel size distribution. After perfusion with μAngiofil^®^, a novel polymerizing contrast agent, low- and high-resolution scans (voxel side length: 2.58–0.66 μm) of the entire vasculature were acquired. Based on the microCT data, sites of interest were defined and samples further processed for correlative morphology. The solidified, autofluorescent μAngiofil^®^ remained in the vasculature and allowed co-registering of the histological sections with the corresponding microCT-stack. The perfusion efficiency of μAngiofil^®^ was validated based on lectin-stained histological sections: 98 ± 0.5% of the blood vessels were μAngiofil^®^-positive, whereas 93 ± 2.6% were lectin-positive. By applying this approach we analyzed the angiogenesis induced by the cell-based delivery of a controlled VEGF dose. Vascular density increased by 426% mainly through the augmentation of medium-sized vessels (20–40 μm). The introduced correlative and quantitative imaging approach is highly reproducible and allows a detailed 3D characterization of the vasculature and muscle tissue. Combined with histology, a broad range of complementary structural information can be obtained.

Occlusive vascular disorders, such as peripheral artery disease (PAD) and critical limb ischemia, are major global health issues, causing vascular morbidity and mortality. In 2010, a total of 202 million patients throughout the world were suffering from PAD^1^. Therapeutic angiogenesis is a potential treatment strategy to modulate the microcirculation and therefore ameliorate ischemic conditions in those patients. Particularly, vascular endothelial growth factors (VEGFs), being key regulators of angiogenesis, have been extensively studied as potential drug treatment, although with limited success^2^,^3^,^4^,^5^. Recent studies have focused on investigating vascular alterations caused by the application or discontinuation of angiomodulating therapies onto diseased and healthy tissues^6^,^7^.

The murine hind limb is one of the most widely used preclinical models to study therapeutic angiogenesis. This is mainly due to the anatomical similarities with the human limb and its relatively simple microvascular architecture with most capillaries running in parallel to the muscle fibres. The current gold standard for quantifying skeletal muscle’s microvasculature is assessing capillary density and capillary-to-fiber ratio based on histological cross-sections. However, histological approaches have several significant limitations. Besides shrinkage and tissue distortion occurring during sample preparation, this technique does not provide any information about the three-dimensional (3D) vascular architecture or the vascular pattern, unless serial sectioning is performed. Serial sectioning, however, is very laborious, time-consuming, and the precise alignment is often delicate^8^,^9^. Vascular corrosion casts examined by scanning electron microscopy is another tool commonly used to study the microvasculature^10^. Although it provides images from superficial vessels, deeper vessels inside the sample cannot be visualized and quantitative information can barely be extracted. Similar limitations also apply to earlier studies investigating the skeletal muscle microcirculation by transillumination, and thus being restricted to thin muscles (e.g., murine spinotrapezius or cremaster, hamster retractor, rat gracilis and rabbit and cat tenuissimus muscles)^11^,^12^. The microvasculature of thicker muscles like the ones of the murine hind limb could not be completely visualized with sufficient detail resolution. In short, there is a considerable demand for high-resolution vascular 3D imaging techniques, which allow visualizing the microvasculature of thicker muscles.

3D imaging techniques like micro-computed tomography (microCT) have been receiving increasing attention in recent years. Despite providing volumetric data at near-microscopic resolution, the vasculature cannot be discriminated from other soft tissues on the basis of its inherent x-ray attenuation. Thus, the vascular perfusion with a radiopaque contrast agent is indispensible for visualizing the vasculature by microCT^13^. In vascular research, microCT combined with various perfusion-based contrast agents has been used to visualize the vasculature of various organs, such as the heart, the liver, kidneys, lungs, the hind limb but also of tumors^14^,^15^,^16^,^17^,^18^,^19^,^20^. However, those studies were limited in terms of resolution, size of volume of interest, filling and perfusion of the vasculature (also due to the relatively high viscosity of the applied perfusion-based contrast agent)^13^,^17^ and, therefore, restricted to the detection of vessels sized 20–50 μm in the diameter. In a recent publication by Ehling J. and coworkers^20^. The authors stated that “blood vessels as small as 3.4 μm …could be visualized…”. Unfortunately, declared isotropic voxel sizes ranging from 3.4 to 5.3 µm do not allow the unambiguous identification of such small vessels - not even in theory.

Vascular corrosion casting followed by tissue maceration provides better detectability and has been successfully used for microCT-visualization of capillaries in a tiny subsample, e.g., an individual glomerulus (scanned at an isotropic voxel side length of 1 µm)^21^. Although several groups have demonstrated the feasibility of visualizing capillaries in skeletal muscle when using vascular corrosion casts, to our knowledge, no one has yet succeeded in visualizing capillaries *in situ* by microCT, without prior tissue maceration^21^,^22^,^23^,^24^.

In this study, we introduce a correlative *ex vivo* imaging approach, which allows investigating the murine hind limb vasculature and its surrounding tissue from the whole organ to the capillary level. To visualize the vasculature, we used the novel polymerizing vascular contrast agent μAngiofil^®^, which provides a sufficient strong signal to depict the vasculature including capillaries. In addition, to visualize the muscle fibers, the tissue was dehydrated and covered with a thin layer of paraffin. Finally, the tissue was further processed for histology and/or immunohistochemistry, which enabled a detailed characterization of the microvasculature of any region of interest. In order to exemplify the utility of this approach, we applied it to analyze the angiogenic effects induced by the local overexpression of a controlled dose of Vascular Endothelial Growth Factor-A (VEGF) in skeletal muscle, taking advantage of a well-characterized cell-based gene delivery platform^25^,^26^.

## Methods

For a detailed description of the experimental methods, please see the Materials and Methods section of the Online Data Supplement.

### Myoblast implantation

To study the local effects of VEGF overexpression, a previously described monoclonal population of primary murine myoblasts was implanted in three hind limb muscles of SCID CB.17 mice, as previously described^27^,^28^. The animals were housed and sacrificed according to the Swiss Animal Welfare Act and Swiss Animal Welfare Ordinance. All experimental protocols were approved either by the veterinary office of the canton of Bern with the license number BE27/12 or the veterinary office of the canton of Basel-Stadt with the license number 2071. In total 11 mice were used in the study (n = 6 for cell implantation and n = 5 for establishing the method/non-injected control).

### Sample preparation

#### For the visualization of the vasculature

Deeply anaesthetized mice were injected with μAngiofil^®^ (Fumedica AG, Muri, AG, Switzerland), a polymerizing vascular contrast agent, through the descending aorta. After polymerization, hind limbs were collected, fixed in 2% paraformaldehyde and stored until scanning.

#### For the visualization of the muscle fiber architecture

Samples were prepared as described above. Before scanning, hind limbs were decalcified over 4 days in 10% EDTA (pH 7.5), dehydrated and covered with a thin layer of paraffin.

### MicroCT imaging

Samples were scanned using a desktop microCT (SkyScan 1172 or 1272, Bruker, MicroCT, Kontich, Belgium). MicroCT projections were back projection-reconstructed using the NRecon software (NReconServer64bit, Bruker, MicroCT, Kontich, Belgium) and volume-rendered and visualized in 3D with the CTVox software (Bruker, MicroCT, Kontich, Belgium). Muscle tissue and blood vessels were segmented and analyzed using the CTAn software (Bruker, MicroCT, Kontich, Belgium). Blood vessel sizes were assessed using Matlab (The MathWorks, Inc., Natick, MA, USA) and plotted using Excel (Microsoft Corporation, Redmond, WA, USA).

### Histology, immunohistochemistry and immunofluorescence

After microCT scanning, decalcified hind limbs or single muscles were further processed for histology. 5 μm-thick paraffin-sections were prepared and stained either with Azan trichrome, Masson trichrome, BS-1 Lectin (L3759, Sigma Aldrich Co., St. Louis, MO, USA) or with a monoclonal anti-slow skeletal myosin heavy chain antibody [NOQ7.5.4D] (Abcam, Cambridge, UK). Azan and Masson trichrome were chosen to facilitate the comparability between histology and microCT: both stainings highlight the connective tissue (appearing in blue), which also appears lighter colored in x-ray projections (higher x-ray attenuation than muscle tissue).

### Perfusion efficiency

Perfusion efficiency was assessed based on lectin-stained histological sections, where μAngiofil^®^-perfused and non-perfused vessels were determined. Using a systematic random sampling procedure 4 fields of view were selected per section and 3 sections per muscle. Therein, a total of 1265 ± 88 capillaries per animal were evaluated by classifying each capillary either as μAngiofil-positive/Lectin-positive (μAF+/L+), μAngiofil-positive/Lectin-negative (μAF+/L−), or μAngiofil-negative/Lectin-positive (μAF−/L+).

### Density maps

Density maps representing the number of capillaries per area (capillary density) and the capillary-to-fiber ratio were generated. For the former one, blood vessels were manually identified in the digital image and analyzed using an in-house written Matlab-script. For each kernel (squared area) the blood vessels were summed up and the sum (number of blood vessels contained in a kernel) was eventually divided by the area of the kernel (in μm^2^). For the latter one, the capillary-to-fiber ratio within a pre-defined area (kernel) was determined and the density map was generated.

## Results

### Visualization of blood vessels and muscle fibers/muscle fiber bundles

**Figure 1:**
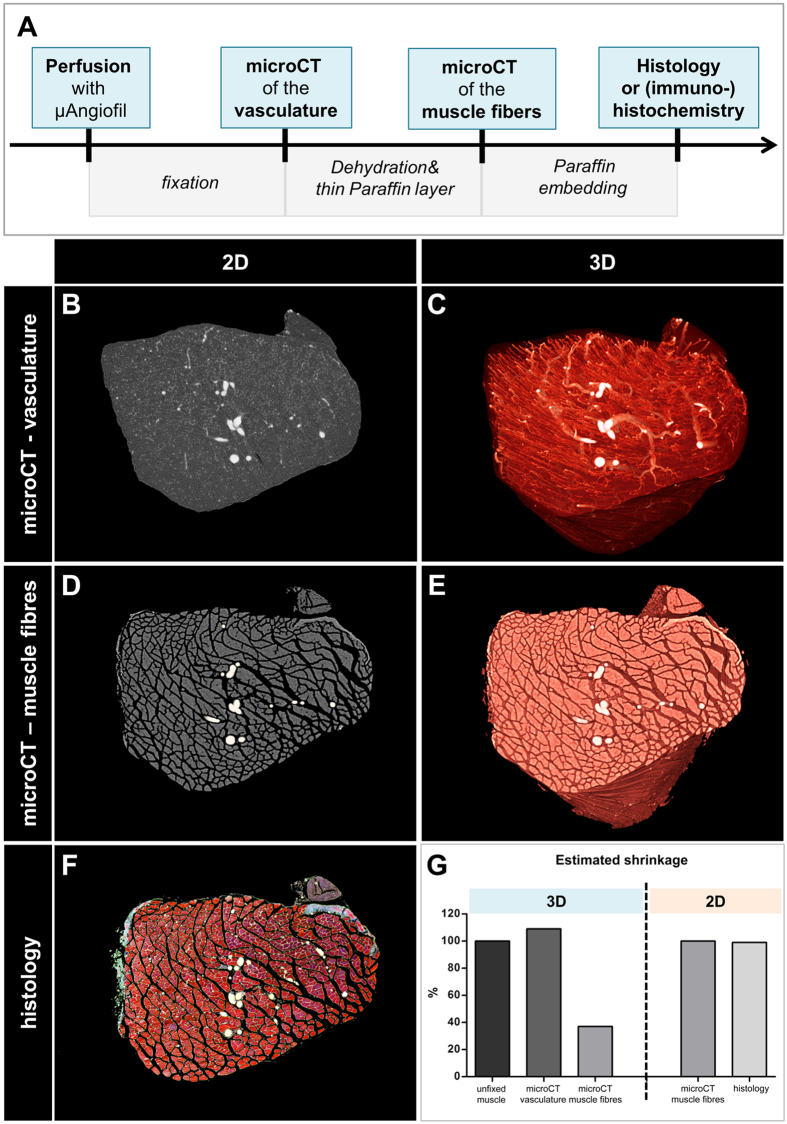
Correlative imaging approach to visualize the vasculature and fiber arrangement by microCT and histology. (**A**) Workflow. (**B**,**C**) Contrast-enhanced vasculature of plantaris muscle in 2D (**B**) and 3D (**C**). Voxel side length: 0.99 μm. (**C**,**D**) Muscle fibers of the same plantaris muscle in 2D (**C**) and 3D (**D**). Voxel side length: 1.39 μm. (**F**) Corresponding histological cross-section stained with Masson trichrome. (**G**) Estimated shrinkage between the different steps of the procedure. Larger blood vessels were manually highlighted in white.

For the consecutive imaging of blood vessels and their surrounding muscle tissue we established a multi-stage procedure (Fig. 1A). First, the blood was removed from deeply anaesthetized and heparinized mice. Thereafter, approximately 3 ml of the dark blue μAngiofil^®^ were injected into the circulation via the descending aorta. As soon as μAngiofil^®^ polymerized, hind limbs were collected, muscles were dissected, fixed and stored (for days or weeks) until being scanned.

The first microCT-scan was performed in order to visualize the contrast-enhanced vasculature (Fig. 1B,C). In a next step, the muscle was prepared similar to the standard procedure for histology: it was dehydrated in an ascending alcohol series, and then transferred to xylene substitute before being infiltrated by paraffin. However, instead of embedding the muscle into a paraffin-block, only a thin layer of paraffin was left around the muscle. At this point, a second microCT-scan was performed to delineate muscle fibers (Fig. 1D,E). The most compelling scanning parameters that were used for the different applications were summarized in Table 1. Lastly, the muscle was embedded for histology, sectioned and stained (Fig. 1F). This three-step protocol used microCT to provide 3D morphological information of the vasculature and the musculoskeletal system and standard immunohistochemistry to generate complementary 2D information at the microscopic level.

At each of these steps, tissue shrinkage occurred to a different extent (Fig. 1G). The relative shrinkage at each of the aforementioned steps was assessed by comparing microCT-derived volume measurements (relative volume shrinkage) or muscle area measurements (relative area shrinkage). From the unfixed muscle to its fixed state (=microCT-scan of the vasculature) no volume shrinkage was observed (109%), whereas from the fixed to the dehydrated/paraffinized state (=microCT-scan of the muscle fibers) considerable shrinkage of 63% was measured. The area shrinkage between the dehydrated and the sectioned state (=histology) was 1%. Consequently, it was not possible to directly co-register blood vessels and muscle fibres; still, they can be co-registered regarding their level and obliquity. Although isotropic shrinkage occurred, no significant tissue distortion was noticed, suggesting that the morphological structures kept their relationship to each other.

### Musculoskeletal system of the murine hind limb

**Figure 2:**
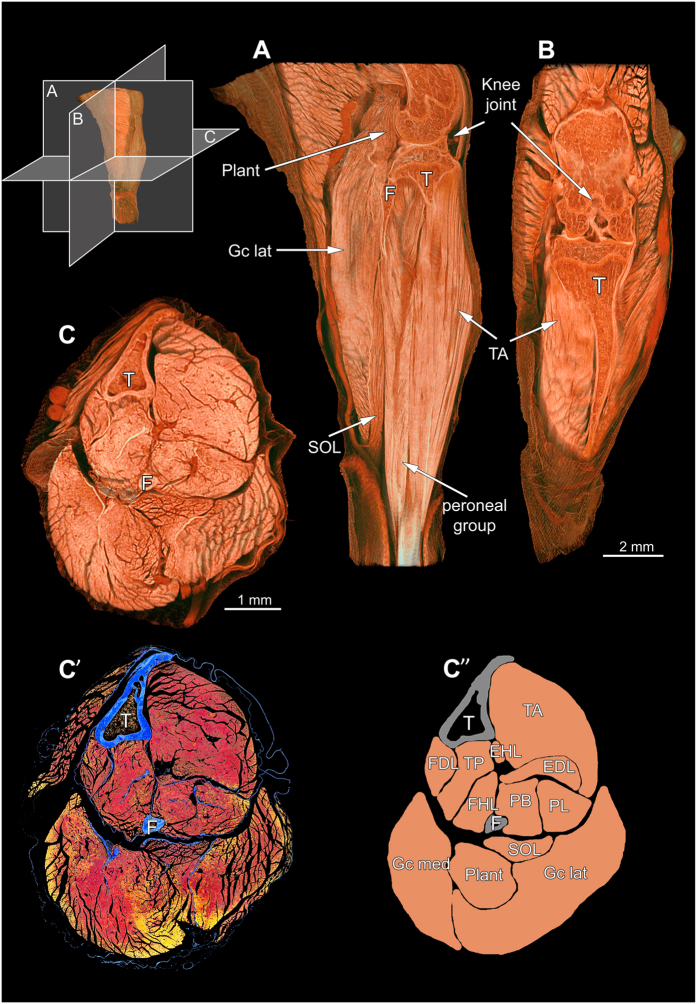
Musculoskeletal system of the left murine lower hind limb. (**A**) Lateral, (**B**) Sagittal and (**C**) Transversal view through the hind limb imaged by microCT. Voxel side length: 2.99 μm. (**C’**) Corresponding histological cross-section stained for Azan Trichrom (blue: bones and connective tissue, red-yellow: muscle tissue. (**C”**) Scheme with labelled muscles. T = tibia, F = fibula, TA = tibialis anterior, EDL = extensor digitorum longus, EHL = extensor hallucis longus, PB = peroneus brevis, PL = peroneus longus, FDL = flexor digitorum longus, TP = tibialis posterior, FHL = flexor hallucis longus, SOL = soleus, Plant = plantaris, Gc med = gastrocnemius medialis, Gc lat = gastrocnemius lateralis.

For a better orientation within the murine hind limb, we performed an overview scan of the entire musculoskeletal system. After 3D reconstruction, virtual sections through the entire hind limb could be obtained at any given height and in any given plane (sagittal, longitudinal or transversal), without destroying the sample (Fig. 2A–C *and online video S1*). Because connective tissue components showed slightly higher x-ray attenuation than the muscle fibers themselves, individual muscles were readily identifiable. Even at a relatively low resolution (isometric voxel side length: 2.99 μm), muscle fibers bundles were distinguishable and their orientation and fiber architecture including pennation angles could be clearly delineated. Moreover, since the sample remained intact, it was further processed for histology (Fig. 2C’).

### Contrast-enhanced microvasculature of the murine hind limb visualized by microCT

**Figure 3:**
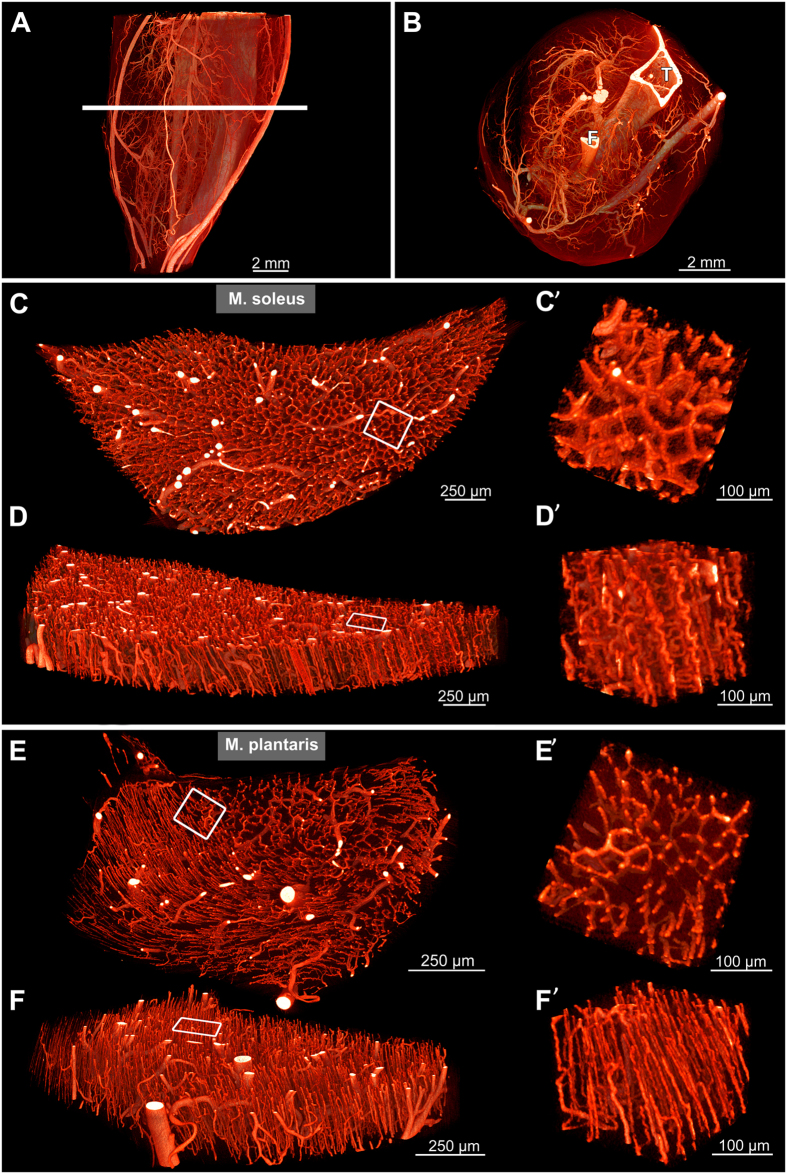
Vasculature of the murine lower hind limb visualized by microCT. (**A**) Lateral 3D view of the hind limb vasculature. Voxel side length: 2.7 μm. (**B**) Virtual transverse section of the vasculature (at the level indicated in **A**). Tibia (T) and fibula (F) appear lightly colored due to their high x-ray absorption. (**C–F**) Virtual transverse sections of isolated soleus (**C**,**D**) and plantaris (**E**,**F**) muscle, respectively. Voxel side length: 0.8 and 0.66 μm respectively. (**C’–F’**) Volumes of interest indicated by rectangles in (**C–F**), showing volume-rendered microvasculature at higher magnification with differing vessel densities, tortuosity and 3D arrangement.

In order to study the vascular network of the murine hind limb in 3D, a multi-scale imaging approach was followed. First, an overview scan of the contrast-enhanced vasculature was acquired (Fig. 3A,B). This enabled the visualization of all blood vessels 10 μm in diameter or larger. The intravital injection of the solidifying contrast agent enabled an accurate visualization of the vasculature *in situ* and preserves the spatial relationship to other morphological structures, such as bones and nerves. Two muscles, soleus and plantaris, known to exhibit different fiber type characteristics, were chosen to further characterize their microvascular architecture. Both muscles were scanned at higher resolution (voxel side length: 0.8 resp. 0.66 μm) to detect all blood vessels including capillaries (Fig. 3C–F). Based on virtual transverse sections of the two muscles, equally sized volumes of interest were selected in order to study the vascular pattern in more detail (Fig. 3C’–F’). At higher magnification, the differences in vascular pattern between different muscles became more evident. In soleus muscles, capillaries appeared highly tortuous and built a dense vascular network surrounding the muscle fibers (Fig. 3C’,D’ *and online video S2*), whereas in plantaris muscles, capillaries appeared straighter, more sparsely arranged and less organized (Fig. 3E’,F’).

This multi-scale top-to-bottom imaging approach provides 3D information on the microvasculature and, at the same time, enables the maintenance of an overall view of the entire hind limb.

### Perfusion efficiency of μAngiofil^®^

**Figure 4:**
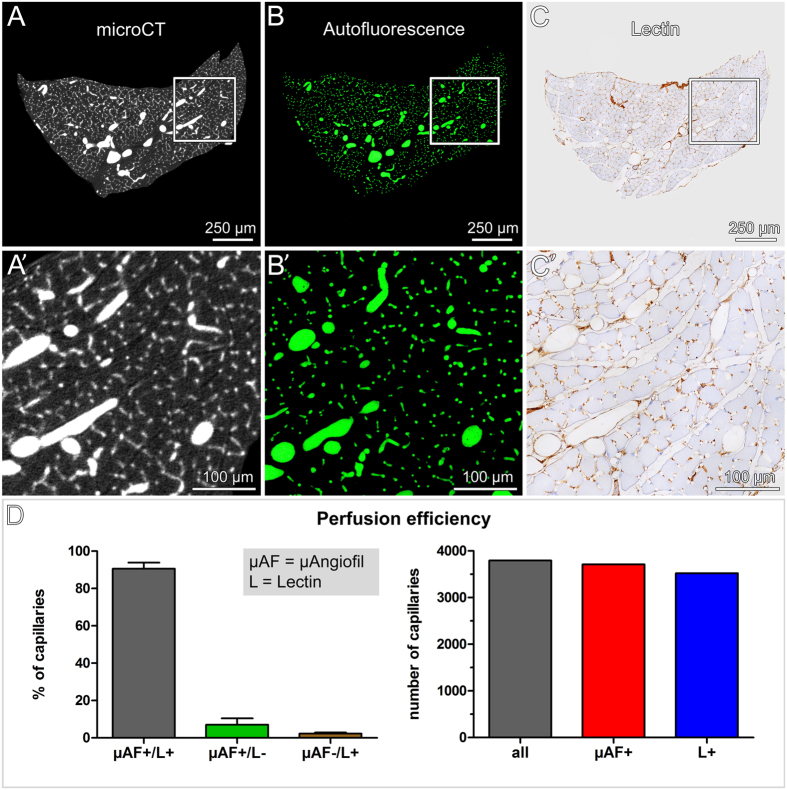
Validation of microCT data by standard histology. (**A**) Virtual cross-section of the vasculature of soleus muscle obtained by high-resolution microCT. Voxel side length: 0.8 μm. (**B**,**C**) Corresponding histological cross-sections. Vessels filled with μAngiofil^®^ (green) (**B**) and subsequent section stained for lectin (brown) (**C**). (**A’**,**B’**,**C’**) Region of interest, indicated with squares, at higher magnification. (**D**) Perfusion efficiency (n = 3).

To assess the perfusion efficiency of μAngiofil^®^, scanned muscles were further processed for histology. Serial sections were prepared and imaged at high magnification. Because of the μAngiofil^®^’s strong inherent fluorescent signal, perfused blood vessels were detected without prior vessel staining. Subsequently, lectin-stained histological sections were manually registered across the entire microCT dataset to find their corresponding virtual sections. The comparison of these section pairs, showed an excellent agreement between the two imaging modalities (Fig. 4A,B). In a next step, the perfusion efficiency of μAngiofil^®^ was quantitatively assessed comparing μAngiofil^®^-induced auto-fluorescence with lectin staining (Fig. 4B,C). A total of 3800 capillaries were evaluated (Fig. 4D). 91% (±3%) of all capillaries were μAngiofil^®^- & lectin-positive, 2% (±0.5%) were only lectin-positive, whereas 7% (±3%) were only μAngiofil^®^-positive. μAngiofil^®^ allowed an adequate perfusion and visualization of the entire vasculature including the smallest capillaries.

### Heterogeneity of skeletal muscle capillarization

**Figure 5:**
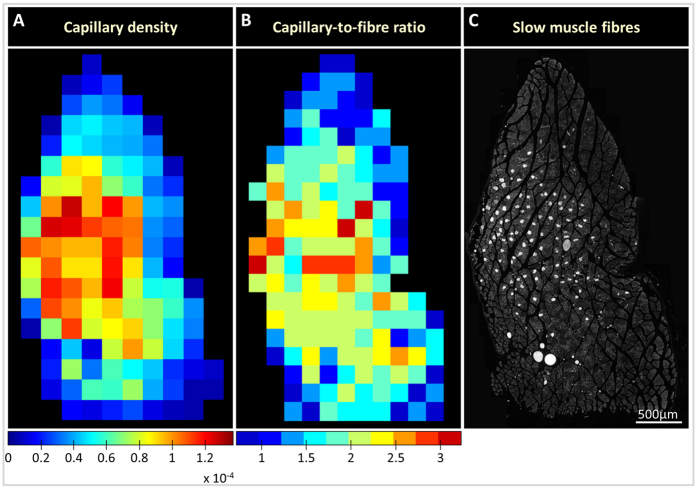
Capillary density correlates with slow muscle fiber distribution in the medial gastrocnemius muscle. (**A**) Density maps representing number of capillaries per area (capillary density) (**A**) and capillary-to-fiber ratio (**B**). (**C**) Slow muscle fibers distribution revealed by anti-slow skeletal myosin heavy chain antibody.

Skeletal muscle is composed of different muscle fiber types which can be classified into slow and fast-contracting muscle fibers^29^. While some muscles display a rather homogeneous mosaic of fiber types, others display enormous regional heterogeneities. Consequently, capillaries are not evenly distributed throughout such muscles. Thus, two density maps based on histological images were generated to evaluate the spatial distribution of capillaries throughout gastrocnemius medialis muscle because it is known to be composed of a mixture of slow- and fast-twitch fibers^30^,^31^ (Fig. 5). Figure 5A shows a “conventional” density map representing the number of capillaries per area, whereas Fig. 5B displays the relative distribution of capillaries and fibers (capillary-to-fiber ratio). Both, capillary density and capillary-to-fiber ratio distribution indicated a highly vascularized deep muscle portion and a sparsely vascularized superficial portion. As expected, this correlated well with the distribution of slow muscle fibers, which were stained using an anti-slow skeletal myosin heavy chain antibody (Fig. 5C).

### Effects of site-specific delivery of VEGF-transfected myoblasts

**Figure 6:**
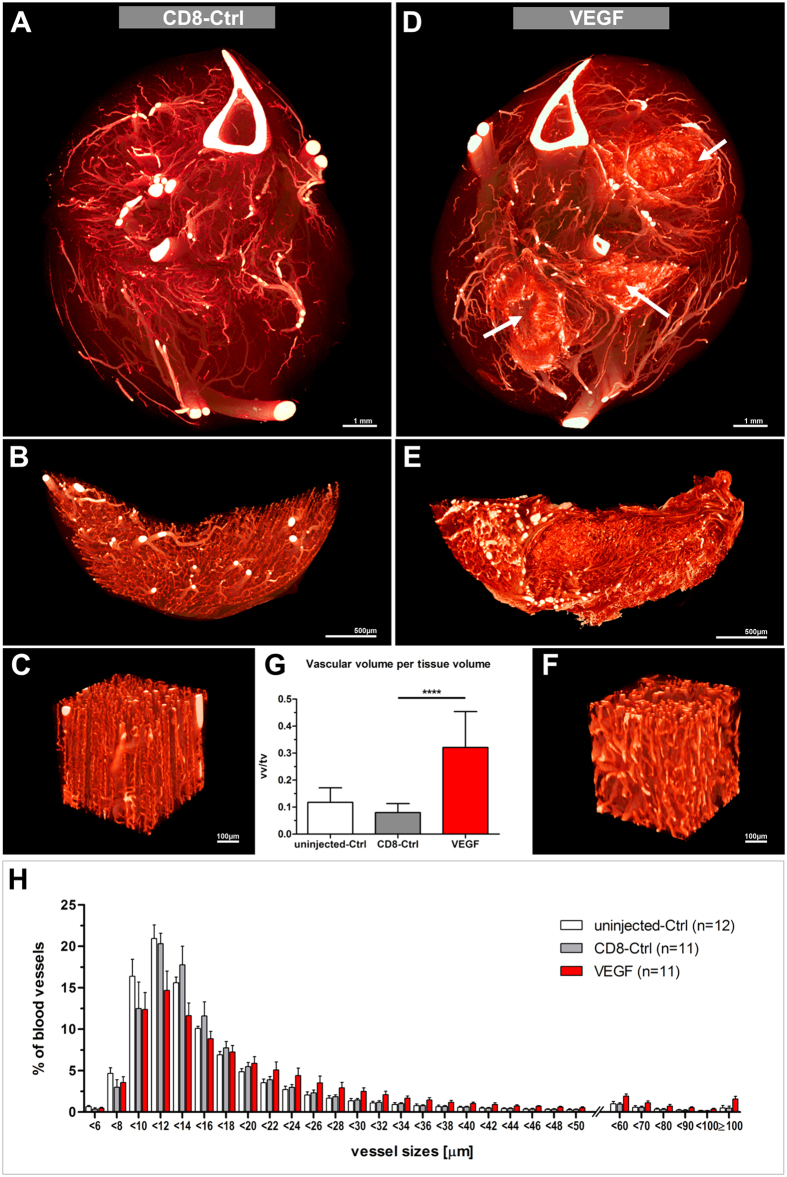
Therapeutic application of VEGF-transduced myoblasts. (**A**,**D**) Vascular microCT-scans of CD8-control (**A**) and VEGF-treated (**D**) contralateral hind limbs respectively (voxel side length: 2.58 μm). VEGF-injection sites are indicated by arrows. (**B–F**) High-resolution microCT-scans of the soleii muscles (**B**,**E**) and regions of interest at higher magnification (**C**,**F**) (voxel side length: 0.92 μm). (**G**) Vascular volume measurements. Vascular volume (vv) per tissue volume (tv) within a given injection or control site. ****p < 0.0001. (**H**) Vessel size distribution. Histogram presenting the relative proportion of a given vessel size. Vessel diameters are given in μm. Measurements were conducted on 20 equidistant virtual sections per sample.

We sought to further validate this novel vascular imaging approach by performing a 3D analysis of VEGF–induced vascular growth. For this purpose, we took advantage of a previously well-characterized model of controlled VEGF gene delivery for therapeutic angiogenesis in skeletal muscle. This platform relies on monoclonal myoblast populations retrovirally transduced to over-express specific and homogeneous levels of VEGF, allowing a localized, sustained and controlled VEGF-overexpression with no systemic effects^26^,^27^,^28^. A myoblast clone homogeneously secreting a moderate amount of VEGF, which was previously shown to robustly induce only normal and therapeutically effective angiogenesis^25^,^32^, was chosen and implanted into 3 hind limb muscles of immunodeficient SCID mice (n = 6). CD8^+^-control-myoblasts (CD8-Ctrl) were injected into the contralateral hind limb instead. Since it was previously shown that networks of normal and mature capillaries are fully formed 1 week post implantation of the VEGF-expressing myoblasts^25^, hind limbs were collected and scanned using microCT 10 days post implantation. Based on the overview scans (voxel side length: 2.58 μm), no significant vascular effects could be detected in any of the control hind limbs (Fig. 6A,D), whereas the three injection sites of VEGF-transduced myoblasts could be clearly identified and localized, and envelopes of densely arranged vessels could be seen surrounding each injection site (Fig. 6D *and online video S3*).

As expected, vascular volume measurements of the injection sites indicated robust vascular growth in the VEGF-treated muscles compared to CD8-Ctrl and uninjected muscles (0.32 ± 0.13 vs. 0.08 ± 0.03 and 0.12 ± 0.05, p < 0.0001) (Fig. 6G). Muscles treated with CD8-control cells did not show any vascular changes compared to the non-treated control muscles.

In order to obtain a more detailed view of the injection sites and their surrounding tissue, the soleii muscles were carefully isolated. The high-resolution scan (voxel side length: 0.92 μm) revealed a dense vascular network comprising tortuous and enlarged blood vessels, but with a regular morphology, in close vicinity to the injection site (Fig. 6E,F). Quantification of vessel size distribution (Fig. 6H) confirmed an increase in diameter in the VEGF-treated muscles compared to the control muscles (15.7 ± 1.7 (Median_VEGF_) vs. 13.4 ± 0.5 (Median_CD8_) and 12.9 ± 0.6 (Median_uninjected_)). As anticipated^27^,^33^, in this area immediately adjacent to the injection site no increase in capillary-size structures could be measured, which are known to be induced exclusively in the microenvironment around VEGF-expressing cells^26^. However, specifically medium-sized vessels larger than 20 μm were increased, which was compatible with the previously described arteriologenic effect of VEGF^27^,^33^.

## Discussion

### MicroCT-based visualization of the skeletal muscle vasculature

In this study, we present an *ex vivo* microCT imaging approach, in combination with the novel polymerizing contrast agent μAngiofil^®^, as a versatile and widely applicable tool for vascular-related research. Although microCT has been frequently used to evaluate arteriogenesis/collateral vessel formation, no one has yet succeeded in visualizing the vasculature in its entirety, down to the capillary level^34^,^35^,^36^,^37^.

To our knowledge, we are the first to present microCT-based visualization of skeletal muscle’s vasculature down to capillary level without prior tissue maceration. This microCT approach provides detailed tomographic 3D data at near-microscopic resolution, thereby providing new insights into the microvascular architecture, its hierarchical structure and geometrical complexity, such as branching pattern, anastomoses and tortuosity. As demonstrated, the capillaries within soleus muscle are much more tortuous and build more anastomoses than the ones in plantaris muscle. Few researchers considered the heterogeneous distribution of blood vessels within muscles when evaluating the vasculature by classical histology. To overcome potential sampling bias, which could lead to erroneous results, we suggest quantifying vascular volumes instead.

Furthermore, this data could be particularly useful for the creation of computational models to improve simulations on blood flow and shear stress as well as facilitate progress in the field of microvascular network analysis. While earlier studies dealing with microvascular network analysis were mainly restricted to the analysis of flat and easily accessible tissue samples and/or thin muscles of the animals that could be transilluminated^11^. The here presented approach provides 3D-datasets of significantly thicker tissue samples enabling a qualitatively new level of microvascular network analysis.

### Benefits

#### Manpower and time efficiency

Current approaches for 3D visualization of blood vessels include serial sectioning or vascular corrosion casting, both of which are destructive, time-consuming and entail various difficulties. Unlike these methods, microCT allows time-efficient 3D imaging without loss of any sections. Taken together, sample preparation (30 min), fixation (overnight), image acquisition (4 h), reconstruction (approx. 4 h), post-processing (2 h) and generation of the final 3D image, all can be performed within 2 days.

#### Correlative morphology

MicroCT is a non-destructive imaging technique, so upon scanning, the chemically fixed tissue is still amenable for further analyses by light (or electron) microscopy. Therefore, we developed a correlative imaging approach, combining microCT with subsequent histology. This approach provides considerably more information than “blind” serial sectioning and enables us to visualize structures from different levels of organization, from whole organ down to cellular level. Furthermore, since the used immunohistochemical protocol yielded good results, we do not see any apparent obstacles for the whole-mount staining. It makes our suggested approach even more promising by enabling comparison of multiple 3D imaging modalities. Altogether, complementary information of any region of interest can be extracted and gathered in a very effective and reliable way.

#### μAngiofil^®^ - a new generation contrast agent

The microCT-based visualization of blood vessels relies heavily on the contrast agent used to enhance the otherwise low inherent contrast of blood vessels. Most of the available perfusion-based contrast agents are based on either iodine or heavy metals put in suspension. High toxicity, clumping and vessel obstructions, inhomogeneous signal and disconnected vessels are common issues that have been described^24^. In this study, we used the recently developed perfusion-based contrast agent μAngiofil^®^, which has proven to be an excellent contrast enhancer with extraordinary perfusion properties, yielding a homogeneous signal throughout the tissue specimen. It enters and fills even smallest blood vessels, thus ensuring adequate perfusion of the entire microvascular network. Moreover, it solidifies and retains the vessel morphology but remains elastic enough to allow for easy post-dissection of the tissue of interest. Furthermore, samples were stored in 2% PFA over several months before being scanned and the contrast remained stable. Hence, as long as the samples are not physically destroyed through sectioning, they can be rescanned almost any time. Another convenient feature of μAngiofil^®^ is its inherent fluorescence, which renders tedious capillary staining for fluorescence microscopy unnecessary.

### Limitations

#### Perfusion pressure

Similar to other perfusion techniques, special care should be taken to keep the perfusion pressure as uniform as possible across the different animals. Still, the pressure probably exceeded the physiological one, which could lead to unwanted vessel dilation or leakage. By gross examination we did not find any vessel leakage in any of the non-treated mice and blood vessel sizes were in the normal range of what had been described in the literature^10^. Nevertheless, absolute values for vessel diameters must be interpreted with caution.

#### Tissue shrinkage

Another critical issue, usually underestimated or neglected, is tissue shrinkage occurring during sample preparation for microCT imaging. Only few studies have addressed this important issue^9^,^38^,^39^. Consistent with what had been described previously, we observed an initial swelling of the muscle which was then followed by substantial shrinkage mainly during the dehydration with the increasing alcohol series^38^. Similarly, Buytaert *et al*. found volume shrinkage of nearly 60% after having applied an ethanol-based staining procedure to enhance skeletal muscle’s contrast^40^. Despite this, corresponding sections could be easily identified suggesting that shrinkage occurred homogeneously throughout the sample without any significant deformation.

#### Post-mortem microCT application

The reported imaging modality is designed exclusively for post-mortem analyses and does not allow dynamic 3D monitoring of vascular alterations *in vivo* (longitudinal studies). Although *in vivo* microCT-scanners are constantly improving, the repeated x-ray irradiation exposure and the low spatial resolution remain yet unsolved problems. MicroCT provides purely structural information, and does not provide any functional data. There are other imaging modalities that could provide some functional data, such as Laser Doppler imaging, ultrasound imaging (microbubbles) or contrast echocardiography, positron emission tomography (PET) or single-photon emission computed tomography (SPECT)^41^,^42^,^43^. However, the spatial resolution of these imaging technologies is still poor, sensitivity and penetration depth limited.

#### Data storage

Further limitations of high-resolution 3D imaging include the tremendous amount of data that is generated, especially when high-resolution scans of multiple segments are needed. Hence, image storage, transfer, display and analysis are major challenges and require very powerful computers.

In spite of these drawbacks, contrast-enhanced microCT is a relatively simple and straightforward tool with a broad application range for studying physiological and pathological alterations of the microvasculature of skeletal muscle or also other organs. In addition, pro- and antiangiogenic effects of potential novel therapeutic treatment strategies can be reliably evaluated, qualitatively as well as quantitatively, as illustrated with our study of VEGF-induced vascular growth.

## Additional Information

**How to cite this article:** Schaad, L. *et al*. Correlative Imaging of the Murine Hind Limb Vasculature and Muscle Tissue by MicroCT and Light Microscopy. *Sci. Rep.* **7**, 41842; doi: 10.1038/srep41842 (2017).

**Publisher's note:** Springer Nature remains neutral with regard to jurisdictional claims in published maps and institutional affiliations.

## Supplementary Material

Supplementary Material

Supplementary Video 1

Supplementary Video 2

Supplementary Video 3

## Figures and Tables

**Table 1:** Sample preparation and scanning parameters.

	Tissue	Fixation	Pretreatment	MicroCT parameters					Scan duration	MicroCT	Visualization of
				*kV*	*uA*	*rotation step [°]*	*filter*	*voxel side length [μm]*			
**Vasculature**											
	hind limb	2% PFA	none	59	167	0.25	no	2.85	8h20 (6)*	1172	vasculature
	single muscle	2% PFA	none	49	200	0.15-0.23	no	0.66-0.8	4h40 (1)	1172	microvasculature incl. capillaries
**Musculoskeletal system**											
	hind limb	2% PFA	10% EDTA	40	250	0.25	no	2.99	8h30 (6)	1172	muscle fiber bundles connective tissue
	single muscle	2% PFA	none	49	198	0.25	no	1.39	2h15 (3)	1172	
**Application**											
	hind limb	2% PFA	none	49	200	0.25	Al 0.5	2.58	17h36 (6)	1172	effect of VEGF- and control myoblasts
	single muscle	2% PFA	none	49	198	0.25	no	0.92	3h38 (1)	1172	
	hind limb	2% PFA	none	70	142	0.1	Al 0.5	2.00	10h24 (4)	1272	for quantification

*Enclosed in brackets you find the number of segments that were scanned.
