# Supplementary material for: Correlative Imaging of the Murine Hind Limb Vasculature and Muscle Tissue by MicroCT and Light Microscopy
Source: Sci Rep. 2017 Feb 7;7:41842. doi: 10.1038/srep41842 (PMC5294414; doi:10.1038/srep41842)
Supplement: Supplementary Material [file srep41842-s1.docx]

***Supplemental Material***

**Correlative Imaging of the Murine Hind Limb Vasculature and Muscle Tissue by MicroCT and Light Microscopy**

**Schaad, MicroCT Skeletal Muscle Vasculature**

Laura Schaad*, Ph.D. ^1, 2^

Ruslan Hlushchuk*, M.D. ^1^

Sébastien Barré, Ph. D. ^1^

Roberto Gianni-Barrera, Ph. D. ^3^

Andrea Banfi, M. D. ^3^

Valentin Djonov, Prof., M. D. ^1^

* These authors contributed equally to this work.

Affiliations:

^1^ *Institute of Anatomy, University of Bern, Baltzerstrasse 2, 3012 Bern, Switzerland*

*^2^ Graduate School for Cellular and Biomedical Sciences, University of Bern, Switzerland*

*^3^ Department of Biomedicine, University Hospital Basel, Hebelstrasse 20, 4031 Basel, Switzerland*

Corresponding author:

Valentin Djonov

Institute of Anatomy

University of Bern

Baltzerstrasse 2

3000 Bern 9

Fax: +41 (0)31 631 38 07

Tel: +41 (0)31 631 84 32

djonov@ana.unibe.ch

**Detailed Methods**

*Animals*

For establishing the method, we used C57BL/6J mice (n = 5, male, 3 months old) purchased from Janvier Labs (Le Genest Saint Isle, France). VEGF-overexpressing myoblasts were applied to immunodeficient SCID CB.17 mice (n = 6, 2-3 months old) (Charles River Laboratories, Sulzfeld, Germany).

All mice were housed at 21 °C with a 12h/12h light-dark cycle and had access to food (standard laboratory chow) and water *ad libitum*. All experimental protocols were approved either by the veterinary office of the canton of Bern with the license number BE27/12 or the veterinary office of the canton of Basel-Stadt with the license number 2071.

*Myoblast implantation*

To study the local effects of VEGF overexpression, a previously described monoclonal population of primary mouse myoblasts, which homogeneously expressed a moderate VEGF level and induced only normal angiogenesis, was implanted in three hind limb muscles (tibialis anterior, soleus and gastrocnemius medialis muscle) of SCID CB.17 mice, as previously described[^1^](#_ENREF_1). Briefly, myoblasts were transduced with a bicistronic retrovirus co-expressing murine VEGF_164_ and a truncated version of CD8a (trCD8a) as a convenient cell surface marker, or only trCD8a as control (= CD8-Ctrl), as previously described[^2^](#_ENREF_2). Cells were cultured in 5% CO_2_ on collagen-coated dishes, with a growth medium consisting of 40% F10, 40% DMEM low glucose (Sigma-Aldrich Chemie GmbH, Steinheim, Germany) and 20% fetal bovine serum (HyClone, Logan, UT), supplemented with 2.5 ng/ml basic fibroblast growth factor (FGF-2) (Becton Dickinson, Basel, Switzerland), as described[^3^](#_ENREF_3). Myoblasts were dissociated in trypsin, resuspended in sterile PBS with 0.5% BSA and 10 µl containing 1x10^6^ cells were implanted into the aforementioned muscles using a syringe with a 30-gauge needle.

*Sample preparation for microCT*

Contrast-enhanced visualization of the vascular network

Mice were deeply anaesthetized (using fentanyl 0.05 mg/kg body weight (b. w.), midazolam 5 mg/kg b.w., and medetomidin 0.5 mg/kg b.w., i. p.) and immediately injected with 50 µl heparin (i. p.). The thorax was opened and the aorta was exposed. A catheter was carefully inserted into the descending aorta, manually fixed and the right atrium slightly incised to permit drainage of blood. Then, mice were perfused with PBS (37 °C, 40 ml) to remove the blood.

µAngiofil® (Fumedica AG, Muri, AG, Switzerland) was prepared according to the manufacturer’s instructions and then injected through the previously placed catheter until leakage from the heart was observed. Thereafter, it was left to polymerize for 1 h at room temperature. Hind limbs or muscles were collected and immersion-fixed in 2 % paraformaldehyde-PBS until scanning.

Visualization of the musculoskeletal system

Samples were prepared as described above to visualize the vasculature. After being stored in 2 % PFA-PBS, the hind limb was decalcified in 10 % Ethylenediaminetetraacetic acid EDTA (pH 7.5) (Sigma Aldrich Co., St. Louis, MO, USA) over four days, whereby the EDTA-solution was exchanged daily. Thereafter, the samples were dehydrated in ascending alcohol concentrations (from 70-100 % EtOH) and left in Xylene substitute (Sigma Aldrich Co., St. Louis, MO, USA) overnight. The next day, the samples were immersed in paraffin (3 h), before they were covered by a thin layer of paraffin and let air-dry.

*Image acquisition by microCT*

The samples were placed in a pipette tip and fixed with a liquid-saturated sponge to prevent the sample from drying out and from moving during image acquisition. Subsequently, the sample was scanned using desktop microCT (SkyScan 1172 or 1272, Bruker, MicroCT, Kontich, Belgium)(Table 1). After scanning, the tissue was stored in 2 % PFA-PBS at 4 °C until further processing.

*Reconstruction and visualization of microCT data*

MicroCT projections were reconstructed using the NRecon software (NReconServer64bit, Bruker, MicroCT, Kontich, Belgium). The reconstructed datasets (up to 7200 virtual slices of 4904 x 4904 pixels, 22.9 MB, grey scale, 8-bit) were volume-rendered and visualized in 3D with the CTVox software (Bruker, MicroCT, Kontich, Belgium).

*Blood vessel and muscle volume quantification*

For all quantification, scans with identical scanning parameters and an voxel side length of 2.00 µm were used. The CTAn software (Bruker, MicroCT, Kontich, Belgium) was used to segment, quantify, and characterize blood vessels within the injection and control sites. Injection sites could be easily identified and were manually contoured to define our volume of interest (VOI). This VOI was then applied to VEGF-treated (VEGF-overexpressing myoblasts), CD8-control (CD8-control-myoblasts) and uninjected control hind limbs. New datasets were built comprising the injection sites or the corresponding control sites, respectively. Subsequent analyses were all performed on these datasets.

First of all, the tissue volume (tv) contained in the VOI was determined. To do so, images were binarized by using a low threshold and a “3D analysis” was performed. Secondly, the vascular volume (vv) within the VOI was determined. To binarize the images, a threshold, containing all blood vessels and minimal background voxels, was selected empirically. Again, the “3D analysis” was performed.

To assess blood vessel sizes an in-house written Matlab-script (The MathWorks, Inc., Natick, MA, USA) was used. The quantification was carried out based on 20 equidistant 2D images throughout the entire dataset. The Matlab-script used the same threshold value as before to binarize the images. By applying various functions of the “Image Processing Toolbox” of Matlab, all white regions were grouped as objects.

For each object, the vessel diameters were determined. The distance between the two most distant laying pixels within the vessel cross-sectional area was defined as the major axis, whereas the longest distance orthogonal to the respective major axis was defined as the minor axis or vessel diameter respectively. Objects with diameters smaller than 5 µm, were considered non-capillaries and excluded from all analyses. The relative proportion of the different vessel sizes was plotted using Excel (Microsoft Corporation, Redmond, WA, USA).

*Density maps*

To illustrate blood vessel distribution within different muscle portions, two kinds of density maps were generated using an in-house written Matlab-script. Blood vessels were manually segmented. All information of the segmented blood vessels (pixel) contained in a subregion (kernel) was added and divided by the kernel area (in µm^2^). As a result a new image is created, in which all kernels are displayed with the value corresponding to the blood vessel density. A second density map representing the capillary-to-fiber ratio distribution was manually generated by determining the capillary-to-fiber ratio (C:F) within each kernel. The maximal and minimal C:F values were used to set the range of the color code with 10 equidistant steps in-between.

*Shrinkage estimation*

Shrinkage was estimated based on volume measurements of a single muscle in his unfixed, fixed and dehydrated/paraffinized state. To do so, microCT-scans were acquired at each step of the imaging protocol and the muscle volume was calculated using the CTAn software (“3D analysis”). Since the temperature within the microCT-scanner may reach up to 30 °C, scan duration was minimized to prevent dehydration-induced shrinkage. To assess shrinkage occurring between the second mircoCT scan (dehydrated/paraffinized state) and histology (sectioned state), the muscle area was quantified based on point counting a histological section and on the corresponding virtual microCT section.

*Histology*

Azan trichrome: The decalcified murine hind limb was embedded in paraffin and sectioned (5 µm thick sections). Sections were deparaffinized, rehydrated and transferred into aniline alcohol for 45 min. Thereafter, sections were treated with acidulated alcohol and stained with Azocarmine G for 1 h at 56°C. After having rinsed the sections in distilled water, they were differentiated with aniline alcohol for 10 min and treated with acidulated alcohol before being transferred to phosphotungstic acid for 2 min. Sections were then rinsed with distilled water and stained with aniline blue for 1 h. After being rinsed in distilled water, they were again treated with phosphotungstic acid for 5 min. Then sections were first rinsed in distilled water, then in acidulated water for 2 min and quickly in 70 % EtOH. Finally, sections were dehydrated, cleared and mounted.

Masson Trichrome: Paraffin sections were deparaffinized and rehydrated, before being post-fixed by Bouin for 60 min at 60 °C. Sections were rinsed under running tap water, quickly in distilled water and in 95% EtOH. The nuclei were then stained in Weigert’s iron hematoxylin for 5 min. After rinsing again in tap water, sections were transferred into the Masson staining solution (Fuchsine acid and Ponceau Xylidine) for 30 min. After rinsing in 1 % acetic acid, sections were then transferred into 1 % Phosphomolybdic acid for 5 min. Again sections were rinsed in 1 % acetic acid and transferred to 2% Methyl blue for 5 min. After a last rinsing in 1 % acetic acid and distilled water, sections were dehydrated and mounted.

*Histochemistry and Immunofluorescence*

Prior to dehydration and embedding, the samples were washed in 70 % ethanol, which facilitated sectioning of the perfused tissue. Paraffin-sections of 5 µm thickness were prepared, deparaffinized, rehydrated and stained as follows:

Slow muscle fibers type were stained using an anti-slow skeletal myosin heavy chain antibody. After a pre-treatment with 0.1 % (w/v) Pronase (Roche Diagnostics, Rotkreuz, Switzerland) for 20 min at 37 °C, tissue sections were blocked with 5 % (v/v) normal goat serum and incubated overnight at 4 °C with monoclonal anti-slow skeletal myosin heavy chain antibody [NOQ7.5.4D] (Abcam, Cambridge, UK) diluted 1:50 in antibody diluent (Dako, Agilent Technologies, Santa Clara, CA, USA). After washing in PBS, sections were incubated in secondary antibody AlexaFluor 488-labelled anti-mouse IgG_1_ (Invitrogen, Life Technologies, Carlsbad, CA, USA) for 1 h at room temperature. Tissue sections were washed and mounted using Fluoromount (Sigma Aldrich Co. St. Louis, MO, USA). Fluorescent images were taken using a fluorescent microscope (Leitz DMRBE, Leica Microsystems CMS GmbH, Wetzlar, Germany) equipped with a F-View camera (Olympus, Hamburg, Germany) and then merged with Adobe Photoshop CS5 (Adobe Systems Inc., San Jose, CA, USA).

Blood vessels were stained using BS-1 lectin. Deparaffinized tissue sections were pre-treated by incubating them firstly, in 5 % (w/v) EDTA (3 days, 37 °C), secondly, in trypsin (Gibco, Life Technologies, Carlsbad, CA, USA) (30 min, 37 °C) and finally in 100 mM CaCl_2_ (30 min, room temperature). Thereafter, biotinylated lectin (L3759, Sigma Aldrich Co., St. Louis, MO, USA) was diluted 1:50 in lectin buffer (100 ml Tris-buffered saline (TBS), 20 mg MgCl_2_, 11 mg CaCl_2_) and applied overnight at 4 °C. The next day, sections were incubated with peroxidase-conjugated avidin-biotin-complex (Vectastain, Vector Laboratories, Burlingame, CA, USA) according to the manufacturer’s protocol. 3, 3'-Diaminobenzidine (DAB, Invitrogen, Life Technologies, Carlsbad, CA, USA) was used as substrate. Tissue sections were counterstained, mounted with Aquatex (Merck kGaA, Darmstadt, Germany) and imaged using a Zeiss Axio Imager M.2 light microscope (Carl Zeiss Microscopy GmbH, Jena, Germany) equipped with a UC50 camera (Olympus, Hamburg, Germany).

*Perfusion efficiency*

To assess the perfusion efficiency of µAngiofil®, µAngiofil®-perfused and non-perfused vessels were determined. To do so, soleus, plantaris and tibialis anterior muscles of three animals were chosen and further processed for histology. Histological sections of three levels (proximal-, mid- and distal portion) were prepared and stained for lectin (as previously described). Using a systematic random sampling procedure, 1265 ± 88 capillaries per animal (4 fields of view per section and 3 sections per muscle) were evaluated.

*Statistical analysis*

For statistical analyses we used Prism v5.04 (GraphPad Software Inc., La Jolla, CA, USA). Unless otherwise stated, all results are presented as mean ± standard deviation. For single comparisons we used a two-tailed Student’s t-test. For multiple comparisons we used a 1-way analysis of variance (ANOVA) with a Bonferroni post-test. p < 0.05 was considered statistically significant.

**Supplemental References**

1. Gianni-Barrera R, Burger M, Wolff T, Heberer M, Schaefer DJ, Gurke L, Mujagic E, Banfi A. Long-term safety and stability of angiogenesis induced by balanced single-vector co-expression of pdgf-bb and vegf164 in skeletal muscle. *Sci Rep*. 2016;6:21546

2. Misteli H, Wolff T, Fuglistaler P, Gianni-Barrera R, Gurke L, Heberer M, Banfi A. High-throughput flow cytometry purification of transduced progenitors expressing defined levels of vascular endothelial growth factor induces controlled angiogenesis in vivo. *Stem Cells*. 2010;28:611-619

3. Banfi A, Springer ML, Blau HM. Myoblast-mediated gene transfer for therapeutic angiogenesis. *Methods Enzymol*. 2002;346:145-157

**Legends for Video files**

**S1. Video showing the muscle fiber architecture**

The muscle tissue of the murine hind limb imaged by microCT (voxel side length: 2.99 µm).

**S2. Video showing the microvasculature of soleus muscle**

A virtual section through the microvasculature of soleus muscle visualized by high-resolution microCT (voxel side length: 0.8 µm).

**S3. Video showing the VEGF injection sites**

Vascular effects induced by the local overexpression of VEGF in three hind limb muscles, visualized by microCT (voxel side length: 2.58 µm).
